# Cysteine-Altering NOTCH3 Variants Are Associated with an Increased Risk of Autoimmune Diseases

**DOI:** 10.3390/jcm12196278

**Published:** 2023-09-29

**Authors:** Emily Rieder, Jiang Li, Juan L. Rodriguez-Flores, Muhammad Taimur Malik, Vida Abedi, Ramin Zand

**Affiliations:** 1 Geisinger Commonwealth School of Medicine, Geisinger Health System, Scranton, PA 18510, USA; emily.rieder@duke.edu; 2 Department of Pediatrics, Duke University, Durham, NC 27705, USA; 3 Department of Molecular and Functional Genomics, Weis Center for Research, Geisinger Health System, Danville, PA 17822, USA; jli@geisinger.edu (J.L.); vidaabedi@gmail.com (V.A.); 4 Regeneron Genetics Center, Regeneron Pharmaceuticals, Inc., Tarrytown, NY 10591, USA; juan.rodriguezflores@regeneron.com; 5 Neuroscience Institute, Geisinger Health System, Danville, PA 17822, USA; drtaimurmalik@gmail.com; 6 Department of Public Health Sciences, College of Medicine, The Pennsylvania State University, Hershey, PA 17033, USA; 7 Department of Neurology, College of Medicine, The Pennsylvania State University, Hershey, PA 17033, USA

**Keywords:** CADASIL, autoimmune, inflammation, NOTCH

## Abstract

Autoimmune conditions have been reported among patients with cysteine-altering *NOTCH3* variants and CADASIL. This study aimed to investigate the occurrence of autoimmune illnesses and markers of inflammation in such populations. Cases were identified who had a *NOTCH3* cysteine-altering variant from the Geisinger MyCode^®^ Community Health Initiative (MyCode^®^). We further performed external validation using the UK Biobank cohort. A cohort of 121 individuals with a *NOTCH3* cysteine-altering variant from MyCode^®^ was compared to a control group with no non-synonymous variation in *NOTCH3* (*n* = 184). Medical records were evaluated for inflammatory markers and autoimmune conditions, which were grouped by the organ systems involved. A similar analysis was conducted using data from the UK Biobank (*n*~450,000). An overall increase in inflammatory markers among participants with a *NOTCH3* cysteine-altering variant was observed when compared to an age- and sex-matched MyCode^®^ control group (out of participants with laboratory testing: 50.9% versus 26.7%; *p* = 0.0047; out of total participants: 23.1% versus 10.9%; *p* = 0.004). Analysis of UK Biobank data indicated any autoimmune diagnosis (1.63 [1.14, 2.09], *p*= 2.665 × 10^−3^) and multiple sclerosis (3.42 [1.67, 6.02], *p* = 9.681 × 10^−4^) are associated with a *NOTCH3* cysteine-altering variant in any domain. Our findings suggest a possible association between *NOTCH3* cysteine-altering variants and autoimmune conditions.

## 1. Introduction

Panarteritis nodosa-like changes were found in the arteries of patients with a known *NOTCH3* pathogenic variant and CADASIL (cerebral autosomal dominant arteriopathy with subcortical infarcts and leukoencephalopathy) [1,2]. Additionally, markers of inflammation, including positive antinuclear antibodies (ANA), antiphospholipid antibodies, or oligoclonal bands, have been found in the serum of patients with a *NOTCH3* pathogenic variant [3,4,5,6].

It has been hypothesized that interactions between the immune system and the NOTCH3 signaling pathway could be altered by *NOTCH3* pathogenic variants [4]. The *NOTCH3* pathogenic variant seen in CADASIL results in the gain of a novel/toxic function of the mutant NOTCH3 protein [5]. This mutated protein, through altered protein–protein interactions, potentially triggers immune dysregulation and an autoimmune response [5]. It is unclear if this pathway drives the link between CADASIL and the immune system, but *NOTCH3* variants have been associated with both disease pathways independently [7].

Jurynczyk and colleagues conducted an experiment to determine the role of individual NOTCH receptors in the proliferation, cytokine production, and encephalitogenic potential of PLP-reactive T cells. It was discovered that NOTCH3 receptor inhibition in T cells correlated with the downregulation of protein kinase C, suggesting that selective inhibition of the NOTCH3 receptor could play a role in treating autoimmune disorders [7]. Alternatively, this suggests that uncontrolled upregulation of NOTCH3, as seen in CADASIL, could trigger the immune system and lead to an increased incidence of autoimmune diseases.

Although several case studies [1,3,4,8] presented CADASIL associations with autoimmune conditions, a population-wide study has not yet examined this association. This study aimed to investigate the co-occurrence of autoimmune illnesses and markers of inflammation in a population of participants with a *NOTCH3* cysteine-altering variant from the Geisinger MyCode^®^ Community Health Initiative (MyCode^®^). We hypothesize that these participants would present with more frequent markers of inflammation and diagnoses of autoimmune conditions than a control population, suggesting a connection between cysteine-altering *NOTCH3* variants and autoimmune disorders. Furthermore, an external cohort from the UK Biobank was recruited to determine if some autoimmune phenotypes can be validated.

## 2. Materials and Methods

### 2.1. The Geisinger MyCode^®^ Community Health Initiative (MyCode^®^)

Since 2007, individuals have agreed to provide blood and DNA samples for research as part of MyCode^®^. This agreement includes genomic analyses as part of the Regeneron–Geisinger collaboration. Participants are recruited in primary care and specialty clinics throughout the Geisinger Health System (Danville, PA, USA) without regard to underlying diseases. The MyCode^®^ participants’ genetic data is linked to Geisinger electronic health records (EHRs) under a protocol approved by the Geisinger Institutional Review Board (IRB). The participants’ mean age is 57.4 ± 18.1 years (range 2–89), comprising 57.9% women. A total of 97.5% of the participants are Caucasian of European descent. The consent rate to participate in this initiative has been more than 85%. All research was performed in accordance with relevant guidelines/regulations. Informed consent was obtained from all participants and/or their legal guardian(s). The details of enrollment, sample collection, and processing have been previously published [9,10].

### 2.2. Identification of Cases with a NOTCH3cys Variant and Controls in MyCode^®^

The details of our case and control identification process have been previously published [11]. In summary, all variants located in exons 2–24 of the *NOTCH3* gene were called through the Genome Analysis Toolkit best practices pipeline and filtered with a genotyping quality of 30, minimum depth of 10, a minimum allele balance of 20, and a minimum quality by the depth of 5 [12]. These exons correspond to the exons encoding the 34 EGFr domains of the NOTCH3 protein. Individuals found to have a missense mutation leading to a cysteine amino acid alteration in one of the 34 EGFr domains of the NOTCH3 protein (amino acid position 40-1273) (http://www.uniprot.org accessed on 28 August 2023) were defined as cases. The control group consisted of 184 randomly selected individuals without any non-synonymous variants in *NOTCH3* exons 2–24, who were age- and sex-matched with the cases. The Geisinger IRB approved the study, and informed consent was waived.

### 2.3. Assessment of Inflammatory Markers of Autoimmune Diseases in Cases and Controls

EHRs were blinded for *NOTCH3* variant status and were reviewed. Laboratory tests were evaluated for inflammatory markers, including interleukin-6 (IL-6), antinuclear antibodies (ANA), anti-dsDNA, cyclic citrullinated peptide (CCP), rheumatoid factor (RF), ribonucleoprotein antibodies (RNF), SSA-SSB antibodies, antineutrophil cytoplasmic antibodies (ANCA), anti-saccharomyces cerevisiae antibodies (ASCA), anti-tissue transglutaminase antibodies (Anti-tTG), complement, anti-cardiolipin antibodies (ACA), oligoclonal bands, and IgG synthetic rate. Additionally, any autoimmune diagnosis, including systemic lupus erythematosus (SLE), rheumatoid arthritis (RA), inflammatory bowel disease (IBD), chronic kidney disease (CKD), vasculitis, myositis, celiac disease, type 1 diabetes, Hashimoto’s, psoriasis, and multiple sclerosis (MS) in the EHRs was validated. The diagnoses were recorded when the patient had an established diagnosis made by a related specialty and supported by medical records, imaging, and laboratory data. Each chart was reviewed by two blind reviewers (ER and MTM). The third reviewer (RZ) played the role of a tiebreaker.

### 2.4. Assessment of Inflammatory Markers of Autoimmune Diseases in MyCode^®^

We further examined the inflammatory markers in the entire MyCode^®^ (85,580 participants, excluding participants with cysteine-altering *NOTCH3* variants). Logical Observation Identifiers Names and Codes (LOINC) matching were used to identify individuals tested for markers of inflammation. Antinuclear antibodies (ANA), anti-dsDNA, rheumatoid factor (RF), SSA-SSB antibodies, antineutrophil cytoplasmic antibodies (ANCA), and complement tests were recorded. Geisinger billing codes for diagnostics at any encounter setting were mapped to the International Classification of Disease (ICD)-related ICD10 codes. Demographic information was extracted from the structured EHRs.

### 2.5. External Validation-UK Biobank Cohort

The UK Biobank (Regeneron Pharmaceuticals, Tarrytown, NY, USA) was used to identify patients with a missense mutation leading to a cysteine amino acid alteration in one of the 34 EGFr domains of the NOTCH3 protein. Exome- and whole-genome sequencing data from the UK Biobank (*n*~450,000) was queried to identify cysteine-altering *NOTCH3* variants and cysteine-sparing *NOTCH3* variants at the coding region [13]. Clinical data, including lab results for inflammation/autoimmune biomarkers of patients with a cysteine-altering mutation, were compared to a control group of patients who did not have this mutation.

### 2.6. Statistical Analysis

Categorical data are reported as numbers (percentage); continuous data are presented as the mean (SD), as indicated. Statistical comparisons on binary categorical variables between cases and controls were performed using Chi-square and the Fisher exact test. SPSS 26.0 (Chicago, IL, USA) was used for all statistical analyses. Statistical comparisons of binary categorical variables were performed using Fisher’s exact test.

Independent *t*-test, ANOVA, or ANCOVA tests were used for quantitative variables to determine the group difference. Chi-square tests were used for categorical variables to determine the presence of an association between a variable with two categories and an ordinal variable with k categories. For MyCode^®^, the association between the genotype and (endo)phenotypes, including lab values and clinical values, were tested using Firth logistic regression (R package version 4.0.3 “logistf”)(R Foundation, Vienna, Austria), a penalized likelihood-based method to control the small sample bias, particularly in the rare variants association study. Race, sex, and index age, denoted as the last active (last encounter) date, were identified as potential confounding factors and used to adjust the regression.

For UK Biobank data, the burden test (Regenie package) [13] was used to determine the association between the genotype and (endo)phenotypes, including lab values and autoimmune diseases.

An odds ratio with a 95% confidence interval and *p* values were calculated to determine the effect size and significance of the association, if any. Statistical analyses were performed using R, version 3.6.

## 3. Results

A total of 131 participants with a *NOTCH3_cys_* variant were identified. Medical records were available for 121 patients. We evaluated medical records for inflammatory markers and autoimmune diseases against a control group of 184 individuals. The mean age of the cases at the time of their last visit was 58.2 ± 16.9 years, and 38.8% were men. Table 1 includes the demographics and clinical characteristics of the entire cohort. One participant had a *NOTCH3_cys_* EGFr 1–6 variant, and the rest had an EGFr 7–34 variant. The most frequent variant was p.Arg1231Cys (EGFr domain 31), found in 84 individuals. Supplementary Table S1 includes the frequency of *NOTCH3_cys_* variants in MyCode^®^.

### 3.1. Frequency of Inflammatory Markers of Autoimmune Diseases in Cases Versus Age- and Sex-Matched Controls in MyCode^®^

Fifty-five (45.5%) case participants and 75 (40.8%) control participants had previous laboratory testing for inflammatory markers of autoimmune diseases, including antinuclear antibodies (ANA), anti-dsDNA, cyclic citrullinated peptide (CCP), rheumatoid factor (RF), SSA-SSB antibodies, complement, anticardiolipin antibodies (ACA), and antineutrophil cytoplasmic antibodies (ANCA). Out of 55 cases, 28 had a positive result, which indicated a significantly higher frequency compared to the age- and sex-matched controls (out of participants with laboratory testing: 50.9% versus 26.7%; *p* = 0.00466; out of total participants: 23.1% versus 10.9%; *p* = 0.003989) (Table 2). One participant in the case and one in the control had positive oligoclonal bands in cerebral spinal fluid. Ten cases and three controls had more than one inflammatory marker identified in their EHRs. Supplementary Table S2 includes the basic demographics and clinical characteristics of participants tested for inflammatory markers of autoimmune diseases.

### 3.2. Frequency of Autoimmune Diagnoses in Cases Versus Age- and Sex-Matched Controls in MyCode^®^

Autoimmune diagnoses were grouped according to the organ system involved (Table 3). At the time of chart review, thirteen cases had been diagnosed with a group A autoimmune disease versus ten controls (10.7% versus 5.4%) (Table 4). Four cases and four controls had a group B diagnosis (3.3% versus 2.2%). Finally, two cases and two controls had a group C diagnosis (1.7% versus 1.1%). In total, 19 cases and 16 controls had been diagnosed with an autoimmune disease at the time of chart review (15.7% versus 8.7%; *p* = 0.068) (Table 4).

### 3.3. Frequency of Autoimmune Markers and Diseases in the Entire MyCode^®^

Inflammatory markers of autoimmune diseases, including antinuclear antibodies (ANA), anti-dsDNA, rheumatoid factor (RF), SSA-SSB antibodies, complement (C3), and antineutrophil cytoplasmic antibodies (ANCA), were identified in the EHRs of 2104 participants of the 25,984 participants tested for these inflammatory markers in MyCode^®^. This frequency was significantly lower than the cases with a *NOTCH3_cys_* variant (out of participants with laboratory testing: 8.1% versus 50.9%, *p* < 0.00001; out of total participants: 2.5% versus 23.1%, *p* < 0.00001).

### 3.4. Frequency of Autoimmune Diagnoses in Cases with NOTCH3 Mutations in Any EGFR Domain

Autoimmune diagnoses were evaluated and sub-grouped based on *NOTCH3* EGFRs 1–6 versus 7–34 and 1–34. The burden test indicated that any autoimmune diagnosis (1.63 [1.14, 2.09], *p* = 2.665 × 10^−3^) and multiple sclerosis (3.42 [1.67, 6.02], *p* = 9.681 × 10^−4^) are associated with a *NOTCH3* cysteine-altering variant in any domain. The results also indicate a significant association between chronic kidney disease and domains 1–6. (Table 5).

## 4. Discussion

After a thorough chart review of 121 cases with a *NOTCH3_cys_* variant and 184 age- and sex-matched controls, the results indicate an increased frequency of markers of inflammation in the cases versus controls. These results were supported when tested in the entire MyCode^®^, where participants with a NOTCH3 mutation we more likely to have a marker of inflammation present in their serum. In our cohort, subjects with a *NOTCH3_cys_* variant had more autoimmune diagnoses than the age- and sex-matched control group, although the results did not amount to a level of significance. Similar trends were observed analyzing data from the UK Biobank, although multiple sclerosis did show a significant association with NOTCH3 mutations in all domains. Our findings suggest a possible association between *NOTCH3* cysteine-altering variants and immune system dysregulation.

Although this is the first study evaluating the potential connection between *NOTCH3_cys_* mutations and the immune system in a population-based study, several previous studies have hypothesized about this connection. In recent years, the idea of “inflammatory CADASIL” has emerged after observing several cases of CADASIL presenting alongside autoimmune diseases, including multiple sclerosis [3,4,5,8,14]. Although the association can be partially related to misdiagnosis due to some similarities in imaging phenotype, one hypothesis of why MS and CADASIL may co-occur is due to the breakdown of the blood–brain barrier from either direct cerebral micro vessel damage or local breakdown of the endovascular wall due to acute ischemia, allowing exposure of CNS antigens previously unknown by the immune system, initiating a subsequent immune attack [14,15]. Other hypotheses include the mutant NOTCH3 protein inherited in CADASIL leading to immune dysregulation and autoimmunity [4], and uncontrolled inflammation, as seen in autoimmune conditions, leading to increased levels of inflammatory cytokines which induce the activation of NOTCH signaling [7].

In contrast, Broadley and colleagues investigated whether the *NOTCH3* gene was present in known MS patients. This study tested three microsatellite markers (D19S923, D19S1153, and D19S841) flanking the *NOTCH3* gene in 745 simplex MS families. The authors found no known CADASIL-causing mutations in any patients with MS [16]. They suggested that there was no genetic relationship between the *NOTCH3* gene and MS, but they also did not rule out the possibility that patients with an already altered *NOTCH3* gene, as seen in CADASIL, could experience an increase in inflammation or alteration in their immune system functioning.

This study had several strengths, such as leveraging rich longitudinal EHRs and analyzing a genotype-first approach of population-based biobank cohorts. However, the study also has some limitations. First, several of the autoimmune conditions evaluated were not prevalent in the sample. Therefore, it was challenging to determine the significance. Additionally, blood tests are often an unreliable marker for autoimmune disorders. We addressed this by recording diagnoses confirmed by a rheumatologist along with markers of inflammation indicating an autoimmune disease. Results were only significant for increased markers of inflammation in cases versus controls, confirming that autoimmune diagnoses can be subjective. Finally, most participants (97.5%) in MyCode^®^ are Caucasian of European descent. Therefore, the results of this study may not be generalizable to the entire population.

## 5. Conclusions

While our research provides evidence that there may be a link between autoimmune conditions and *NOTCH3_cys_*-altering mutations, further research needs to be performed to better understand this connection and its mechanism. Additionally, if there is an autoimmune mechanism at play in CADASIL patients, therapeutic options should be considered to treat patients with a known *NOTCH3* pathogenic variant who present with signs of inflammation.

## Figures and Tables

**Table 1:** Clinical characteristics and family history in cases with a *NOTCH3*_cys_ variant versus controls.

	Cases (*n* = 121)	Controls (*n* = 184)	*p* Value
Age at last visit, mean (SD)	58.2 (16.9)	57.8 (16.8)	0.87
Men, *n* (%)	47 (38.8)	70 (38.0)	0.90
Stroke, *n* (%)	15 (12.4)	9 (4.9)	0.03
Mild Cognitive Impairment, *n* (%)	1 (0.8)	3 (1.6)	>0.99
Dementia, *n* (%)	7 (5.8)	10 (5.4)	>0.99
Depression, *n* (%)	48 (39.7)	76 (41.3)	0.81
Hypertension, *n* (%)	58 (47.9)	97 (52.7)	0.48
Diabetes, *n* (%)	27 (22.3)	42 (22.8)	>0.99
Current Smoker, *n* (%)	48 (39.7)	74 (40.2)	>0.99
Coronary Artery Disease, *n* (%)	15 (12.4)	49 (26.6)	0.003
Peripheral Vascular Disease, *n* (%)	5 (4.1)	15 (8.2)	0.24
Family History of Stroke, *n* (%)	25 (20.7)	28 (15.2)	0.22
Family History of Dementia, *n* (%)	6 (5.0)	9 (4.9)	>0.99

**Table 2:** Frequency of markers of inflammation present in cases with a *NOTCH3_cys_* variant versus controls tested for the marker (age- and sex-matched).

	Cases (*n* = 39)	Controls (*n* = 59)
**Antinuclear Antibodies, *n* (%)**	12 (30.8)	10 (17.0)
	Cases (*n* = 15)	Controls (*n* = 5)
**Anti-dsDNA, *n* (%)**	0	1 (20.0)
	Cases (*n* = 12)	Controls (*n* = 13)
**Cyclic Citrullinated Peptide, *n* (%)**	2 (16.7)	0
	Cases (*n* = 40)	Controls (*n* = 49)
**Rheumatoid Factor, *n* (%)**	12 (30.0)	7 (14.3)
	Cases (*n* = 16)	Controls (*n* = 12)
**SSA-SSB Antibodies, *n* (%)**	3 (18.8)	2 (16.7)
	Cases (*n* = 10)	Controls (*n* = 8)
**Antineutrophil Cytoplasmic Antibodies, *n* (%)**	1 (10.0)	0
	Cases (*n* = 8)	Controls (*n* = 6)
**Complement, *n* (%)**	3 (37.5)	0
	Cases (*n* = 16)	Controls (*n* = 9)
**Anti-Cardiolipin Antibodies, *n* (%)**	6 (37.5)	3 (33.3)
	Cases (*n* = 1)	Controls (*n* = 1)
**Oligoclonal Bands, *n* (%)**	1 (100.0)	1 (100.0)
	Cases (*n* = 55)	Controls (*n* = 75)
**Any Inflammatory Marker, *n* (%)**	28 (50.9)	20 (26.7) *

* *p* value = 0.00466.

**Table 3:** Classification of autoimmune diseases by the system involved.

Group A Systemic Disease	Group B Gastrointestinal Disease	Group C Neurological Disease
Systemic lupus erythematosus	Inflammatory Bowel Disease	Multiple Sclerosis
Rheumatoid Arthritis	Autoimmune hepatitis	
Sjogren’s Syndrome	Primary biliary cirrhosis	
Psoriasis/psoriatic arthritis		
Mixed Connective Tissue		
CREST/scleroderma		
Polymyositis		
Vasculitis		

**Table 4:** Autoimmune diagnoses in cases with a *NOTCH3_cys_* variant versus controls (age- and sex-matched).

	Cases (*n* = 121)	Controls (*n* = 184)	*p* Value
Group A, *n* (%)	13 (10.7)	10 (5.4)	0.0858
Group B, *n* (%)	4 (3.3)	4 (2.2)	0.717
Group C, *n* (%)	2 (1.7)	2 (1.1)	>0.99
Any Diagnosis, *n* (%)	19 (15.7)	16 (8.7)	0.068

**Table 5:** Autoimmune disease phenotype results lookup in UKB 450 K for *NOTCH3* EGFRs 1–6 versus 7–34 and 1–34.

EGFR Domains	Phenotype Class	Effect (CI)	Pval	AAF	Cases RR|RA|AA	Controls RR|RA|AA
1–6	C reactive protein	−0.22 (−0.1, 0.11)	3.748 × 10^−1^	2.379 × 10^−5^	315,191|15|0	
7–34		0.08 (−0.46, 0.36)	3.776 × 10^−2^ *	9.740 × 10^−4^	314,593|612|1	
1–34		0.07 (−0.01, 0.12)	5.540 × 10^−2^	9.978 × 10^−4^	314,578|627|1	
7–34	Rheumatoid Factor	−0.02 (−0.1, 0.11)	8.454 × 10^−1^	1.315 × 10^−3^	28,432|75|0	
1–34		0 (−0.2, 0.21)	9.936 × 10^−1^	1.333 × 10^−3^	28,431|76|0	
7–34		−0.02 (−0.43, 0.32)	8.335 × 10^−1^	1.308 × 10^−3^	28,972|76|0	
1–34		0 (−0.2, 0.21)	9.780 × 10^−1^	1.325 × 10^−3^	28,971|77|0	
1–6	Autoimmune diagnosis	6.39 (−0.41, 0.32)	1.127 × 10^−2^ *	2.422 × 10^−5^	14,268|3|0	315,974|13|0
7–34		1.56 (1.71, 18.12)	8.296 × 10^−3^ *	9.826 × 10^−4^	14,228|43|0	315,382|604|1
1–34		1.63 (1.14, 2.09)	2.665 × 10^−3^ *	1.007 × 10^−3^	14,225|46|0	315,369|617|1
1–6		6.63 (0.49, 1.59)	1.067 × 10^−2^ *	2.340 × 10^−5^	14,268|3|0	284,835|11|0
7–34		1.55 (1.65, 19.72)	9.105 × 10^−3^ *	9.913 × 10^−4^	14,228|43|0	284,297|548|1
1–34		1.63 (1.12, 2.07)	2.931 × 10^−3^ *	1.015 × 10^−3^	14,225|46|0	284,286|559|1
1–6	Systemic lupus erythematosus (SLE)	0.36 (0.47, 1.54)	8.493 × 10^−1^	2.583 × 10^−5^	468|0|0	328,535|17|0
7–34		0.37 (0, 19,045.1)	3.329 × 10^−1^	9.847 × 10^−4^	468|0|0	327,905|646|1
1–34		0.37 (0.05, 2.81)	3.237 × 10^−1^	1.011 × 10^−3^	468|0|0	327,888|663|1
1–6		0.36 (0.01, 11.91)	8.433 × 10^−1^	2.583 × 10^−5^	490|0|0	328,545|17|0
7–34		0.37 (0, 15821.8)	3.247 × 10^−1^	9.846 × 10^−4^	490|0|0	327,915|646|1
1–34		0.37 (0.05, 2.71)	3.151 × 10^−1^	1.010 × 10^−3^	490|0|0	327,898|663|1
1–6		0.36 (0.02, 8.68)	8.479 × 10^−1^	2.462 × 10^−5^	464|0|0	304,113|15|0
7–34		0.36 (0, 3317.28)	3.182 × 10^−1^	9.948 × 10^−4^	464|0|0	303,523|604|1
1–34		0.36 (0.05, 2.74)	3.094 × 10^−1^	1.019 × 10^−3^	464|0|0	303,508|619|1
1–6	Rheumatoid arthritis (RA)	0.36 (0.01, 16.44)	6.659 × 10^−1^	2.586 × 10^−5^	4527|0|0	324,193|17|0
7–34		1.09 (0, 79.93)	8.042 × 10^−1^	9.856 × 10^−4^	4517|10|0	323,573|636|1
1–34		1.06 (0.57, 2.09)	8.533 × 10^−1^	1.011 × 10^−3^	4517|10|0	323,556|653|1
1–6		0.36 (0.11, 1.17)	6.645 × 10^−1^	2.583 × 10^−5^	4752|0|0	324,283|17|0
7–34		1.13 (0, 97.18)	7.125 × 10^−1^	9.846 × 10^−4^	4741|11|0	323,664|635|1
1–34		1.1 (0.6, 2.12)	7.597 × 10^−1^	1.010 × 10^−3^	4741|11|0	323,647|652|1
1–6	Other rheumatoid arthritis	8.39 (0.12, 1.12)	2.313 × 10^−1^	2.475 × 10^−5^	5851|1|0	297,123|14|0
7–34		1.7 (0.01, 17.35)	7.138 × 10^−2^	9.918 × 10^−4^	5834|18|0	296,555|581|1
1–34		1.62 (0.93, 2.89)	5.182 × 10^−2^	1.017 × 10^−3^	5833|19|0	296,541|595|1
1–6	Vasculitis (VS)	0.37 (0.17, 1.35)	9.587 × 10^−1^	2.562 × 10^−5^	189|0|0	331,548|17|0
7–34		0.37 (0, 1.19118 × 10^21^)	5.189 × 10^−1^	9.827 × 10^−4^	189|0|0	330,914|650|1
1–34		0.37 (0.02, 7.74)	5.175 × 10^−1^	1.008 × 10^−3^	189|0|0	330,897|667|1
1–6		0.37 (2.15, 55.9)	9.579 × 10^−1^	2.583 × 10^−5^	183|0|0	328,852|17|0
7–34		0.37 (0, 4.02358 × 10^20^)	5.266 × 10^−1^	9.846 × 10^−4^	183|0|0	328,222|646|1
1–34		0.37 (0.02, 8.14)	5.251 × 10^−1^	1.010 × 10^−3^	183|0|0	328,205|663|1
1–6	Type 1 diabetes (T1D)	0.35 (2.26, 58.68)	7.728 × 10^−1^	2.562 × 10^−5^	426|0|0	331,311|17|0
7–34		1.18 (0, 568.9)	8.806 × 10^−1^	9.827 × 10^−4^	425|1|0	330,678|649|1
1–34		1.07 (0.14, 9.69)	9.512 × 10^−1^	1.008 × 10^−3^	425|1|0	330,661|666|1
1–6		0.36 (0.01, 14.42)	8.458 × 10^−1^	2.583 × 10^−5^	399|0|0	328,636|17|0
7–34		1.24 (0, 18791.2)	8.482 × 10^−1^	9.846 × 10^−4^	398|1|0	328,007|645|1
1–34		1.17 (0.14, 10.59)	8.832 × 10^−1^	1.010 × 10^−3^	398|1|0	327,990|662|1
1–6	Myositis	0.37 (0.01, 13.18)	9.086 × 10^−1^	2.463 × 10^−5^	259|0|0	304,286|15|0
7–34		1.83 (0, 131617)	6.339 × 10^−1^	9.932 × 10^−4^	258|1|0	303,698|602|1
1–34		1.77 (0.15, 24.32)	6.493 × 10^−1^	1.018 × 10^−3^	258|1|0	303,683|617|1
1–6	Inflammatory bowel disease (IBD)	0.37 (0.01, 22.91)	9.126 × 10^−1^	2.583 × 10^−5^	169|0|0	328,866|17|0
7–34		0.37 (0, 474,178,000)	5.218 × 10^−1^	9.846 × 10^−4^	169|0|0	328,236|646|1
1–34		0.37 (0.02, 7.87)	5.157 × 10^−1^	1.010 × 10^−3^	169|0|0	328,219|663|1
1–6		0.37 (0, 125.92)	9.219 × 10^−1^	2.583 × 10^−5^	179|0|0	328,856|17|0
7–34		0.37 (0, 6,059,030,000)	5.099 × 10^−1^	9.846 × 10^−4^	179|0|0	328,226|646|1
1–34		0.37 (0.02, 7.25)	5.053 × 10^−1^	1.010 × 10^−3^	179|0|0	328,209|663|1
1–6	Chronic kidney disease	10.39 (0, 107.19)	4.177 × 10^−2^ *	2.462 × 10^−5^	3764|1|0	300,871|14|0
7–34		1.66 (0, 84.79)	1.548 × 10^−1^	9.946 × 10^−4^	3753|12|0	300,292|592|1
1–34		1.83 (0.81, 3.24)	8.717 × 10^−2^	1.019 × 10^−3^	3752|13|0	300,278|606|1
1–6	Multiple sclerosis (MS)	24.45 (0.24, 3.04)	1.130 × 10^−2^ *	2.584 × 10^−5^	1425|1|0	327,485|16|0
7–34		3.16 (4.65, 225.96)	3.004 × 10^−3^ *	9.850 × 10^−4^	1417|9|0	326,863|637|1
1–34		3.42 (1.67, 6.02)	9.681 × 10^−4^ *	1.011 × 10^−3^	1416|10|0	326,847|653|1
1–6		24.48 (0.39, 20.69)	1.181 × 10^−2^ *	2.583 × 10^−5^	1462|1|0	327,573|16|0
7–34		3 (4.54, 241)	4.217 × 10^−3^ *	9.846 × 10^−4^	1454|9|0	326,951|637|1
1–34		3.25 (1.58, 5.71)	1.417 × 10^−3^ *	1.010 × 10^−3^	1453|10|0	326,935|653|1

Results of the burden test for NOTCH3 extracellular domain EGFRs 1–6, 7–34, and 1–34 in 450 K UKB participants for autoimmune diseases and autoimmune disease biomarkers. From left-to-right are the domains included in the burden test (1–6, 7–34, or 1–34); the phenotype class; the phenotype name; effect size (with 95% confidence interval in parenthesis); *p* value (with * if less than 0.05); alternate allele frequency; the number of cases split by genotype (reference homozygote|heterozygotes|alternate homozygotes); and the number of controls split by genotype (blank for quantitative traits).

## Data Availability

The data presented in this study are available on request from the corresponding author. The data are not publicly available due to patient privacy.

## Supplementary Materials

The following supporting information can be downloaded at: https://www.mdpi.com/article/10.3390/jcm12196278/s1, Table S1: NOTCH3cys variants in MyCode^®^, Table S2: Clinical characteristics and family history in cases with a NOTCH3cys variant versus controls tested for inflammatory markers of autoimmune diseases.

## References

[1] Rafalowska J., Dziewulska D., Fidzianska A. (2004). CADASIL: What component of the vessel wall is really a target for Notch 3 gene mutations?. Neurol. Res..

[2] Rafalowska J., Fidziańska A., Dziewulska D., Podlecka A., Szpak G.M., Kwieciński H. (2003). CADASIL: New cases and new questions. Acta Neuropathol..

[3] Paraskevas G.P., Bougea A., Synetou M., Vassilopoulou S., Anagnostou E., Voumvourakis K., Iliopoulos A., Spengos K. (2014). CADASIL and Autoimmunity: Coexistence in a Family With the R169C Mutation at Exon 4 of the NOTCH3 Gene. Cerebrovasc. Dis..

[4] Paraskevas G.P., Constantinides V.C., Kapaki E. (2018). Cerebral autosomal dominant arteriopathy with subcortical infarcts and leucoencephalopathy vs. multiple sclerosis. Either one or sometimes both?. Neuroimmunol. Neuroinflamm..

[5] Collongues N., Derache N., Blanc F., Labauge P., de Seze J., Defer G. (2012). Inflammatory-like presentation of CADASIL: A diagnostic challenge. BMC Neurol..

[6] Pantoni L., Sarti C., Pescini F., Bianchi S., Bartolini L., Nencini P., Basile A.M., Lamassa M., Kalaria R.N., Dotti M.T. (2004). Thrombophilic risk factors and unusual clinical features in three Italian CADASIL patients. Eur. J. Neurol..

[7] Jurynczyk M., Jurewicz A., Raine C.S., Selmaj K. (2008). Notch3 Inhibition in Myelin-Reactive T Cells Down-Regulates Protein Kinase Cθ and Attenuates Experimental Autoimmune Encephalomyelitis. J. Immunol..

[8] Khan A., Abedi V., Li J., Malik M.T., Esch M., Zand R. (2020). CADASIL Versus Multiple Sclerosis: Is It Misdiagnosis or Concomitant? A Case Series. Front. Neurol..

[9] Schwartz M.L., McCormick C.Z., Lazzeri A.L., Lindbuchler D.M., Hallquist M.L., Manickam K., Buchanan A.H., Rahm A.K., Giovanni M.A., Frisbie L. (2018). A Model for Genome-First Care: Returning Secondary Genomic Findings to Participants and Their Healthcare Providers in a Large Research Cohort. Am. J. Hum. Genet..

[10] Williams M.S., Buchanan A.H., Davis F.D., Faucett W.A., Hallquist M.L.G., Leader J.B., Martin C.L., McCormick C.Z., Meyer M.N., Murray M.F. (2018). Patient-centered precision health in a learning health care system: Geisinger’s genomic medicine experience. Health Aff..

[11] Hack R., Rutten J., Person T., Li J., Khan A., Griessenauer C., Abedi V., Lesnik Oberstein S.A., Zand R., Regeneron Genetics Center (2020). Cysteine-altering NOTCH3 variants are a risk factor for stroke in the elderly population. Stroke.

[12] McKenna A., Hanna M., Banks E., Sivachenko A., Cibulskis K., Kernytsky A., Garimella K., Altshuler D., Gabriel S., Daly M. (2010). The Genome Analysis Toolkit: A MapReduce framework for analyzing next-generation DNA sequencing data. Genome Res..

[13] Backman J.D., Li A.H., Marcketta A., Sun D., Mbatchou J., Kessler M.D., Benner C., Liu D., Locke A.E., Balasubramanian S. (2021). Exome sequencing and analysis of 454,787 UK Biobank participants. Nat. Res..

[14] Schiess N., Huether K., Szolics M., Agarwal G., El-Hattab A.W., Sathe S. (2018). Multiple sclerosis or “inflammatory CADASIL?”: Case Report and review of the literature. Clin. Neurol. Neurosurg..

[15] Dichgans M., Wick M., Gasser T. (1999). Cerebrospinal fluid findings in CADASIL. Neurology.

[16] Broadley S.A., Sawcer S.J., Chataway S.J.S., Coraddu F., Coles A., Gray J., Roxburgh R., Clayton D., Compston D.A.S. (2001). No association between multiple sclerosis and the Notch3 gene responsible for cerebral autosomal dominant arteriopathy with subcortical infarcts and leukoencephalopathy (CADASIL). J. Neurol. Neurosurg. Psychiatry.

