# Supplementary material for: Cysteine-Altering NOTCH3 Variants Are Associated with an Increased Risk of Autoimmune Diseases
Source: J Clin Med. 2023 Sep 29;12(19):6278. doi: 10.3390/jcm12196278 (PMC10573689; doi:10.3390/jcm12196278)
Supplement: Supplementary file 1 [file jcm-12-06278-s001.zip › jcm-2609480-supplementary.pdf]

Supplemental Tables

Supplemental Table S1. ***NOTCH3*$_{cys}$ variants in MyCode®**

Nucleotide alteration	Protein alteration	Exon	EGFr domain	Frequency in MyCode®	Frequency in gnomAD	Frequency in UK Biobank(18)	Reported in CADASIL pedigrees(19)
c.602G>C	p.Cys201Ser	4	5	1	0	0	n
c.1474A>T	p.Ser492Cys	9	12	1	0	0	n
c.1531T>C	p.Cys511Arg	10	13	1	0	0	y
c.1630C>T	p.Arg544Cys	11	13-14	1	83	2	y
c.1732C>T	p.Arg578Cys	11	14	1	10	2	y
c.1817G>A	p.Cys606Tyr	11	15	1	0	0	n
c.1819C>T	p.Arg607Cys	11	15	4	1	1	y
c.1903C>T	p.Arg635Cys	12	16	4	2	0	n
c.1960T>G	p.Cys654Gly	13	16	1	0	1	n
c.1999G>T	p.Gly667Cys	13	17	1	1	0	y
c.2149C>T	p.Arg717Cys	14	18	2	10	0	y
c.2182C>T	p.Arg728Cys	14	18	2	2	1	y
c.2498T>G	p.Phe833Cys	16	21	1	0	0	n
c.2729G>A	p.Cys910Tyr	17	23	1	0	0	n
c.2747A>G	p.Tyr916Cys	17	23	1	0	0	n
c.2817T>G	p.Cys939Trp	18	24	1	0	0	n
c.3016C>T	p.Arg1006Cys	19	26	1	0	0	y
c.3209G>C	p.Cys1070Ser	20	27	1	0	0	n
c.3298C>T	p.Arg1100Cys	20	28	1	0	0	n
c.3427C>T	p.Arg1143Cys	21	29	6	5	0	y
c.3431A>G	p.Tyr1144Cys	21	29	2	0	0	n
c.3568C>T	p.Arg1190Cys	22	30	7	15	2	n
c.3601C>T	p.Arg1201Cys	22	30	4	10	3	n
c.3691C>T	p.Arg1231Cys	22	31	84	225	33	y
c.3696C>G	p.Cys1232Trp	22	31	1	0	0	n

Supplemental Table S2. Clinical characteristics and family history in cases with a *NOTCH3*$_{cys}$ variant versus controls tested for inflammatory markers of autoimmune diseases.

	Cases (n=55)	Controls (n=75)	P value
Age at last visit, mean (SD)	59.4	56.7	
Men, n (%)	22 (40.0)	22 (29.3)	0.2
Stroke, n (%)	8 (14.5)	3 (4.0)	0.03
Mild Cognitive Impairment, n (%)	1 (1.8)	2 (2.7)	0.75
Dementia, n (%)	2 (3.6)	5 (6.7)	0.57
Depression, n (%)	27 (49.0)	34 (45.3)	0.18
Hypertension, n (%)	26 (47.2)	40 (53.3)	0.49
Diabetes, n (%)	15 (27.3)	21 (28.0)	0.93
Current Smoker, n (%)	18 (32.7)	34 (45.3)	0.15
Coronary Artery Disease, n(%)	6 (10.9)	23 (30.7)	0.008
Peripheral Vascular Disease, n (%)	2 (3.6)	9 (12.0)	0.09
Family History			
Stroke, n (%)	15 (27.3)	10 (13.3)	0.05
Dementia, n (%)	6 (10.9)	2 (2.7)	0.05
